# The results of a study of the causes and correlations between stress and sleep disorders by medical professionals

**DOI:** 10.1192/j.eurpsy.2024.1616

**Published:** 2024-08-27

**Authors:** P. Sarantuya, B. Purev, T. Myatav

**Affiliations:** ^1^medical department, Etugen university; ^2^Avicenna science center, Ulaanbaatar, Mongolia

## Abstract

**Introduction:**

Prolonged exposure to stress can adversely affect mental health and lead to mental illness, which can adversely affect the provision of medical care. It has been determined that sleep disturbances affect physical and mental health and negatively affect daily activities. Therefore, we conducted this study with the assumption that it is an opportunity to improve health care by examining the prevalence of stress in the medical profession and identifying its causes.

**Objectives:**

To study the prevalence of stress and sleep disorders among doctors and medical professionals in Selenge Province General Hospital2. Identify some factors affecting stress and sleep disorders and their relationship

**Methods:**

Using SRQ20, PHQ9, GAD7, and sleep disturbance questionnaires issued by WHO for doctors of primary health care institutions, according to the analytical research model, the ethics committee with the informed consent form, and the research was conducted.

**Results:**

Doctors and medical professionals aged 23-65 participated in the study, the average life expectancy was 37.05 years. 44.44% are stressed. 8% of stressed people had severe stress, 18.89% had no sleep disorder and 81.11% had a sleep disorder. 46.67% of those with sleep disturbances had mild sleep disturbances. But 34.44% had sleep disorders. 30% had a non-organic sleep disorder, 5.56% had lucid dreaming disorder, and 3.33% had non-organic insomnia. According to the correlation analysis, the SRQ20 stress score GAD7 anxiety score is r=0.76, the PHQ9 score is r=0.74, the sleep disturbance score is r=0.68, the satisfaction score is r=-0.44, the sleep disturbance score GAD7 score r=0.75, a moderate positive correlation with the PHQ9 depression score r=0.45, and a weak inverse correlation with the satisfaction score r=-0.24 was related. In the composite linear regression analysis, the stress score increased by 116.2% when the stress problem score increased by one, the anxiety problem score increased by 44.34%, the body shape problem screening questionnaire increased by 82.86%, and the depression problem score increased by one. 73.18% per increase of one, and 7.18% per increase of PHQ9 depression score was statistically significant. On the other hand, the sleep disorder score increases by 127.05% when the stress problem score increases by one, the anxiety problem score increases by 120.79% and the body shape problem detection questionnaire score increases by one.

**Conclusions:**

Doctors and medical professionals need to increase their coping skills, psychiatric examination and diagnosis, and psychological counseling. Also, by implementing the right lifestyle habits, most of the sleep disorders of doctors and medical professionals can be normalized by themselves. Stress is associated with depression, anxiety, sleep disturbances, years of work, relationship satisfaction, psychological problems, and depression.

**Disclosure of Interest:**

None Declared

